# ATGL and CGI-58 are lipid droplet proteins of the hepatic stellate cell line HSC-T6[S]

**DOI:** 10.1194/jlr.M062372

**Published:** 2015-10

**Authors:** Thomas O. Eichmann, Lukas Grumet, Ulrike Taschler, Jürgen Hartler, Christoph Heier, Aaron Woblistin, Laura Pajed, Manfred Kollroser, Gerald Rechberger, Gerhard G. Thallinger, Rudolf Zechner, Günter Haemmerle, Robert Zimmermann, Achim Lass

**Affiliations:** * Institute of Molecular Biosciences, University of Graz, Graz, Austria; † Bioinformatics, Institute for Knowledge Discovery, Graz University of Technology, Graz, Austria; § Institute of Forensic Medicine, Medical University of Graz, Graz, Austria; ** BioTechMed-Graz, Graz, Austria; †† OMICS Center, Graz, Austria

**Keywords:** proteome, lipidome, neutral lipid hydrolase, adipose triglyceride lipase

## Abstract

Lipid droplets (LDs) of hepatic stellate cells (HSCs) contain large amounts of vitamin A [in the form of retinyl esters (REs)] as well as other neutral lipids such as TGs. During times of insufficient vitamin A availability, RE stores are mobilized to ensure a constant supply to the body. To date, little is known about the enzymes responsible for the hydrolysis of neutral lipid esters, in particular of REs, in HSCs. In this study, we aimed to identify LD-associated neutral lipid hydrolases by a proteomic approach using the rat stellate cell line HSC-T6. First, we loaded cells with retinol and FAs to promote lipid synthesis and deposition within LDs. Then, LDs were isolated and lipid composition and the LD proteome were analyzed. Among other proteins, we found perilipin 2, adipose TG lipase (ATGL), and comparative gene identification-58 (CGI-58), known and established LD proteins. Bioinformatic search of the LD proteome for α/β-hydrolase fold-containing proteins revealed no yet uncharacterized neutral lipid hydrolases. In in vitro activity assays, we show that rat (r)ATGL, coactivated by rat (r)CGI-58, efficiently hydrolyzes TGs and REs. These findings suggest that rATGL and rCGI-58 are LD-resident proteins in HSCs and participate in the mobilization of both REs and TGs.

Vitamin A is an essential fat-soluble micro-nutrient in vertebrates (1). Vitamin A is taken up from the body mostly in form of retinol (ROH) or as provitamin β-carotene. The latter is converted to ROH in a two-step process in intestinal mucosa cells (2). ROH is transported in the circulation either unesterified, bound to ROH-binding protein 4 (RBP4), or esterified as retinyl esters (REs) in the hydrophobic core of lipoproteins. Furthermore, REs represent the storage form of vitamin A and are deposited in cytosolic lipid droplets (LDs) of various cell types [for review see (3–5)].

For normo-physiologic function, mammals require small amounts of retinoids (vitamin A and metabolites) for biological activities: 11*-cis*-retinal is the bioactive metabolite in the visual cycle, and 9*-cis* and all-*trans* retinoic acids are the ligands for nuclear receptors which, upon binding, transactivate gene expression (6, 7). Despite the small amounts of vitamin A required for biological activities (e.g., <25 pmol retinoic acid per gram tissue), large amounts of vitamin A (∼1 μmol/g liver) are stored as REs in LDs of specialized liver cells that are known as fat-storing cells, lipocytes, Ito cells, or hepatic stellate cells (HSCs) (8, 9). In mice, for instance, this hepatic vitamin A pool is sufficient to ensure and maintain vitamin A supply for several weeks (10, 11). Interestingly, hepatic vitamin A stores are also mobilized under pathological conditions, e.g., chronic alcoholic injury of the liver (12, 13). However, loss of vitamin A stores upon advanced liver disease (at the stage of liver fibrosis) is basically nonreversible (14–16).

LDs of HSCs not only contain REs but also TGs and cholesteryl esters (CEs) which are surrounded by a phospholipid (PL) monolayer (relative amounts of lipids are ∼40% REs, ∼40% TGs, ∼15% CEs, and ∼5% PLs, which vary depending on nutrition) (17, 18). Neutral glycerolipids have in common that the hydroxyl groups of their backbones are esterified to FAs. The mobilization of these storage lipids requires the hydrolysis of respective ester bonds by specific enzymes, so-called lipases. Several studies have addressed the question of which lipases are expressed and involved in the hydrolysis of neutral lipids in HSCs. Mello et al. (19) reported that adipose TG lipase (ATGL) [annotated as patatin-like phospholipase domain containing protein 2 (PNPLA2)] and LPL are detectable in rat HSCs at the mRNA level. In quiescent HSCs, ATGL mRNA levels were found to be higher than those of LPL. In contrast, in activated HSCs, mRNA levels of LPL increased, while those of ATGL decreased. At the protein level, pancreatic lipase-related protein 2 (PLRP2) and adiponutrin [annotated as patatin-like phospholipase domain containing protein 3 (PNPLA3)] have been shown to be expressed in HSC-T6 cells and human primary HSCs, respectively (20, 21). Silencing of procolipase (Clps) expression, the activator protein of pancreatic TG lipase and PLRP2, increased cellular RE content in HSC-T6 cells (20). Similarly, silencing of adiponutrin in the human cell line, LX-2, increased cellular RE content (21). More recently, Taschler et al. (22) showed that murine ATGL, together with its coactivator comparative gene identification-58 (CGI-58), hydrolyzes both TGs and REs. Interestingly, ATGL-KO mice do not accumulate more REs in the liver than their wild-type littermates. Furthermore, primary HSCs isolated from these mice are capable of mobilizing RE stores, which argues against a limiting role of ATGL in RE mobilization, at least in murine HSCs. Mice globally lacking PLRP2 or adiponutrin have not been reported to show any defect in hepatic RE mobilization. Thus, to date, the identity of the lipase(s) responsible for the mobilization of REs in HSCs is unclear.

One of the reasons why only few studies have attempted to identify lipases of HSCs is the low abundance of HSCs in the liver (5–15% of all liver cells) (8). Hence, the isolation of primary HSCs gives either very low yield or high impurities. The generation of immortalized HSC cell lines, like rat HSC-T6 and human LX-1 and LX-2 cell lines, provide stable, homogenous, and unlimited sources of HSCs. These cell lines have been extensively characterized and have been shown to retain key features of quiescent HSCs (23, 24). Thus, such cell lines can be used to biochemically characterize LDs by lipidomic and proteomic approaches and, more importantly, to identify lipolytic enzymes of the LDs.

In this study, we employed the rat HSC-T6 cell line for a lipidomic and proteomic characterization of HSC-derived LDs. We induced LD formation by loading cells with ROH and oleic or palmitic acid. Lipidomic analysis of LDs revealed that FA composition of TGs and REs shifted toward FA species used for loading of HSC-T6 cells. This was much less evident for CE and PL species. Proteomic analysis of LDs identified a number of well-established LD-associated proteins, such as ATGL, CGI-58, and adipose differentiation-related protein/perilipin 2 (ADRP/PLIN2) with known functions in TG and RE mobilization, respectively. Bioinformatic search for α/β-hydrolase fold-containing proteins in the LD proteome did not reveal additional known neutral lipid hydrolases. In vitro hydrolase activity assays demonstrate that rat (r)ATGL, activated by rat (r)CGI-58, efficiently hydrolyzes both TGs and REs. Because previous studies showed that ATGL-KO mice do not exhibit increased RE stores in the liver, we speculate that an additional, so far unidentified, RE hydrolase must exist, which determines RE content of HSCs.

## MATERIALS AND METHODS

### Materials

Essentially FA-free BSA was obtained from Sigma-Aldrich (St. Louis, MO). The 1,2-diheptadecanoyl-*sn*-glycero-3-phosphatidylcholine (PC), 1,2-dinonadecanoyl-*sn*-glycero-3-PC, triheptadecanoin, and trinonadecanoin were obtained from Larodan (Stockholm, Sweden) and used as internal standards (ISTDs). Retinyl- and cholesteryl-palmitate were purchased from Sigma-Aldrich.

### Cultivation of HSC-T6 cells and loading with ROH and FAs

HSC-T6 cells were obtained from Dr. William S. Blaner, Columbia University of New York. HSC-T6 cells were cultured in DMEM, high glucose (Gibco®; Invitrogen GmbH, Lofer, Germany), supplemented with 10% fetal calf serum and antibiotics at 37°C under humidified atmosphere and 7% CO_2_. To promote LD formation, cells were incubated for 16 h in DMEM supplemented with various concentrations of ROH (5–50 μM; 10 mM stock solution in ethanol) and oleic or palmitic acid (20–200 μM; 4 mM stock solution in PBS, complexed to essentially FA-free BSA in a ratio of 3/1 mol/mol). In some cases, HSC-T6 cells were serum-starved prior to harvest for 2 h by replacing incubation media with serum-free DMEM supplemented with 2% BSA (serum-starvation).

### Isolation of LDs by centrifugation

HSC-T6 cells were collected by trypsinization and resuspended in solution A [250 mM sucrose, 20 mM potassium phosphate (pH 7.0), 1 mM EDTA, 1 mM DTT] containing protease inhibitor mix (20 μg/ml leupeptin, 2 μg/ml antipain, and 1 μg/ml pepstatin). For disruption, the HSC-T6 cell suspension was placed in a 45 ml cell disruption vessel (Parr Instrument Co., Moline, IL) and pressurized with 650 PSI N_2_ gas for 30 min. Then, cell lysate was collected and centrifuged at 1,000 *g* for 5 min at 4°C to remove unbroken cells and nuclei. The supernatant was transferred into a centrifuge tube (Ultra-Clear™ centrifuge tubes; Beckman Instruments Inc.™, Palo Alto, CA), overlaid with solution B [50 mM potassium phosphate (pH 7.4), 100 mM KCl, 1 mM EDTA, protease inhibitor mix), and centrifuged at 100,000 *g* for 1 h at 4°C. The LD layer on the top was collected and used for analyses.

### Assessment of purity of LD preparations by immunoblotting

Proteins of LDs (∼100 μg protein) were precipitated with ice-cold 100% acetone (LD solution/acetone, 1/5, v/v) for 12 h at −20°C, centrifuged at 13,000 *g* for 30 min at 4°C, and brought to dryness. Proteins were dissolved in SDS sample buffer, separated by SDS-PAGE, and transferred onto a polyvinylidene difluoride membrane. The membrane was blocked with nonfat dry milk and probed with antibodies specific for the following marker proteins: GAPDH for cytosol, inositol requiring protein 1α (IRE1α) for microsomes, and PLIN2 for LDs. For detection, the membrane was incubated with horseradish peroxidase-labeled secondary antibodies. Bands were visualized using the ECL Plus Western blotting detection reagent (Thermo Fischer Scientific, Waltham, MA).

### Extraction and quantification of neutral lipids by HPLC

HSC-T6 cells were extracted twice with hexane/isopropanol (3/2, v/v) for 10 min under constant shaking. The organic phases were combined, evaporated, and lipids were dissolved in chloroform/methanol (2/1, v/v). TGs and CEs were separated on a Betasil® diol column (100 × 4.6 mm, 5 μm; Thermo Fisher Scientific) using a ternary gradient solvent system and detected by HPLC-evaporative light scattering detection, as described (25). The HPLC system consisted of a precooled sample manager (at 4°C), pump, injector, and column oven (at 40°C), all of the Agilent 1100 series (Agilent, Santa Clara, CA), and were coupled to a Sedex 85 evaporative light scattering detector (Sedere, Alfortville, France). Data were analyzed using the ChemStation software (B 04.01; Agilent). REs were separated on a YMC-Pro C18 column (150 × 4.6 mm, S-5 μm, 12 nm; YMC Europe GmbH, Dinslaken, Germany) using an isocratic solvent system (98% methanol, 2% water, 1.2 ml/min) and detected at excitation 325 nm/emission 450 nm. The HPLC system consisted of a Waters e2695 separation module, including a column oven (at 35°C) and a Waters 2475 fluorescence detector (Waters Corporation, Milford, MA). Data were analyzed using Empower 3 chromatography data software (Waters Corporation). Neutral lipid standards were prepared as 1 mg/ml stock solutions in chloroform/methanol (2/1, v/v). Calibration curves were measured from 2.7 to 350 μg/ml.

### Separation of neutral lipids by TLC

Extracted lipids were spotted on a silica gel 60 (Merck, Darmstadt, Germany). For comparison, standard solutions containing cholesteryl-palmitate, retinyl-palmitate, and triolein were used. The silica gel was developed using n-hexane/diethylether/acetic acid (80/20/2, v/v/v) as solvent system and lipids were visualized by charring using concentrated sulfuric acid.

### Lipidomic analyses of neutral and PL species of LD preparations

Total lipids of LD preparations (corresponding to 60 μg LD protein including ISTDs) were extracted using chloroform/methanol/water (2/1/0.6, v/v/v). Extraction was performed under constant shaking for 90 min at room temperature. After centrifugation at 1,000 *g* for 15 min at 4°C, the organic phase was collected. Then, chloroform was added to the aqueous phase and extracted as above. Combined organic phases were dried under a stream of nitrogen and dissolved in 1 ml chloroform/methanol/2-propanol (2/1/12, v/v/v) for ultra-performance (UP)LC-quadrupole time of flight (qTOF) analysis. Chromatographic separation was performed using an AQUITY-UPLC system (Waters Corporation) equipped with a BEH-C18 column (2.1 × 150 mm, 1.7 μm; Waters Corporation) as previously described (26). A SYNAPT™ G1 qTOF HD mass spectrometer (Waters Corporation) equipped with an ESI source was used for detection. Data acquisition was done by the MassLynx 4.1 software (Waters Corporation). Lipids were analyzed with the Lipid Data Analyzer 1.6.2 software (27), normalized to ISTDs, and expressed as percent composition.

### SDS-PAGE and LC-MS/MS analysis

For proteomic analysis of LDs, acetone delipidated proteins were separated by SDS-PAGE and stained with Coomassie-brilliant blue. Then, gel lanes were cut into ∼60 slices of approximately equal size. Prior to digestion, individual slices were cut into several 1 mm^2^ pieces. Tryptic digest was performed according to the method by Shevchenko et al. (28). Peptide extracts were dissolved in 0.1% formic acid and separated on an ULTIMATE 3000™ dual gradient nano-HPLC system (Dionex, Amsterdam, The Netherlands). Samples were injected and concentrated on the loading column (PepMap™ C-18, 5 μm 100 Å, 300 μm inner diameter × 1 mm; LC Packings, Amsterdam, The Netherlands) for 5 min using 0.1% formic acid as isocratic solvent at a flow rate of 20 μl/min. The column was then switched into the nano-flow circuit and the sample was loaded on the nano-column (C-18 PepMap™, 75 μm inner diameter × 150 mm, LC Packings) at a flow rate of 300 nl/min and separated using a gradient from 0.3% formic acid and 5% acetonitrile to 0.3% formic acid and 50% acetonitrile over 60 min. Samples were ionized in a Finnigan nano-ESI source (1.5 kV spray voltage) equipped with NanoSpray tips (PicoTip™ Emitter, New Objective) and analyzed in a LTQ iontrap mass spectrometer (Thermo Fisher Scientific).

### Data analyses and bioinformatics

MS/MS spectra were identified by search of the NCBI nonredundant public database (ftp://ftp.ncbi.nih.gov/blast/db/FASTA/) using SpectrumMill Rev. 03.03.084 SR4 software (Agilent). The following search parameters were used: tryptic digestion with one missing cleavage; carbamidomethylation as fixed modification; oxidized methionine, N-terminal pyro-glutamic acid, acrylamide at cysteine as variable modifications; sequence tag length >3; minimum detected peaks of 4; minimum matched peak intensity of 50%; precursor ion mass tolerance of ±2.5 Da, and product mass tolerance of ±0.7 Da. Acceptance parameters were three or more identified distinct peptides with minimum sequence coverage of 20% and probability score of >95% according to Carr et al. (29). Peptide validation and protein clustering were performed with the open access software, MASPECTRAS 2 (30). For peptide filtering, a threshold of 7.5 was used as peptide score and 75% as scored peak intensity score, respectively. Proteins were accepted as identified when at least two peptides passed the aforementioned filters and a total protein score of 25 was reached. Identified proteins were filtered according to: *i*) the plausibility that on the basis of their theoretical molecular mass they could have been identified in the given gel slice; *ii*) the taxonomic classification (*Rattus norvegicus*); and *iii*) their identification in both biological replicates. Identified proteins were grouped based on shared identified peptides, whereas the protein with the highest number of identified peptides was selected. The resulting protein list displays the LD proteome of respective sample sets. These lists were screened for putative hydrolases using the following approaches: *i*) scan of the protein sequences for the GXSXG sequence motif; *ii*) scan for proteins containing the α/β-hydrolase-conserved domain using CDSearch (30, 31); and *iii*) a Reversed Position Specific BLAST (RPSBLAST) scan of the CDD database (version 3.10, filtered for domain entries specific for α/β-hydrolases) (32), to scan for potential α/β-hydrolase domain regions.

### Cloning of recombinant His-tagged rATGL and rCGI-58

The sequences containing the complete open reading frame of rATGL and rCGI-58 were amplified by PCR from rat cDNA using Phusion® high-fidelity DNA polymerase (New England BioLabs Inc., Ipswich, MA). cDNA was prepared from mRNA using SuperScript reverse transcriptase protocol (Invitrogen Life Technologies, Carlsbad, CA). The primers were designed to create endonuclease cleavage sites (underlined) for subsequent cloning strategies: rATGL forward 5′-AAGAATTCATG­TT­CCCAA­GGG­AGACCAAG-3′, rATGL reverse 5′-AAGCGGCC­G­CA­­TCAGC­AA­GGTGGGAGGC­CAGA-3′, rCGI-58 forward 5′-AGAATTCATGAAAGCGATGGCGGCGGA-3′, rCGI-58 reverse 5′-AACTCGAG­TC­AG­TCTACTGTGTGGCAGAT-3′.

PCR products were ligated to compatible restriction sites of the eukaryotic expression vector, pcDNA4/HisMax (Invitrogen Life Technologies). A control pcDNA4/HisMax vector expressing β-galactosidase (LacZ) was provided by the manufacturer (Invitrogen Life Technologies).

### Expression of recombinant proteins and preparation of cell extracts

Monkey embryonic kidney cells (COS-7, ATCC CRL-1651) were transfected using Metafectene (Biontex GmbH, Munich, Germany) as described (33). For the preparation of cell extracts, cells were collected by trypsinization, washed three times with PBS, and disrupted in solution A by sonication (Virsonic 475; Virtis, Gardiner, NY). Nuclei and unbroken cells were removed by centrifugation at 1,000 *g* for 5 min at 4°C. The expression of the His-tagged proteins was detected using Western blotting analysis.

### Determination of neutral lipid hydrolase activities

COS-7 cell lysates containing rat proteins (40 μg in 100 μl) were incubated with various substrates (100 μl) in a water bath at 37°C for 1 h. In some cases, 40 μM Atglistatin (Ai) (in DMSO) or DMSO as control were added. Substrates contained 330 μM triolein and radiolabeled [9,10-3H(N)]triolein (30,000 cpm/nmol) as tracer, respectively. RE substrate contained 300 μM retinyl-palmitate. Substrates were emulsified with either 45 μM (for triolein) or 100 μM (for retinyl-palmitate) PC/phosphatidylinositol (3/1, mol/mol) in 100 mM potassium phosphate (pH 7.4) by repeated sonication (Virsonic 475, SP Scientific Virtis). Then, 5% essentially FA-free BSA was added. For assessment of TG hydrolase activity, radiolabeled FAs were extracted and the amount of radiolabeled FAs was determined by scintillation counting as described (34). Rates of TG hydrolysis were calculated by subtracting counts of blank incubations (100 μl solution A) and normalization to protein. For assessment of RE hydrolase activity, the release of ROH was determined by lipid extraction using 3 ml n-hexane/methanol (1/2, v/v), containing 0.5 mM butylated hydroxytoluene and 100 pmol retinylacetate per sample as ISTD, and ROH content was analyzed by HPLC-fluorescence detection (22).

### Statistical analyses

Data are expressed as means + SD. Statistically significant differences were determined by Student’s unpaired *t*-test (two-tailed). Group differences were considered statistically significant for *P* < 0.05 (*), *P* < 0.01 (**), and *P* < 0.001 (***).

## RESULTS

### Neutral lipid accumulation in HSC-T6 cells upon loading with ROH and FAs

**Fig. 1.**
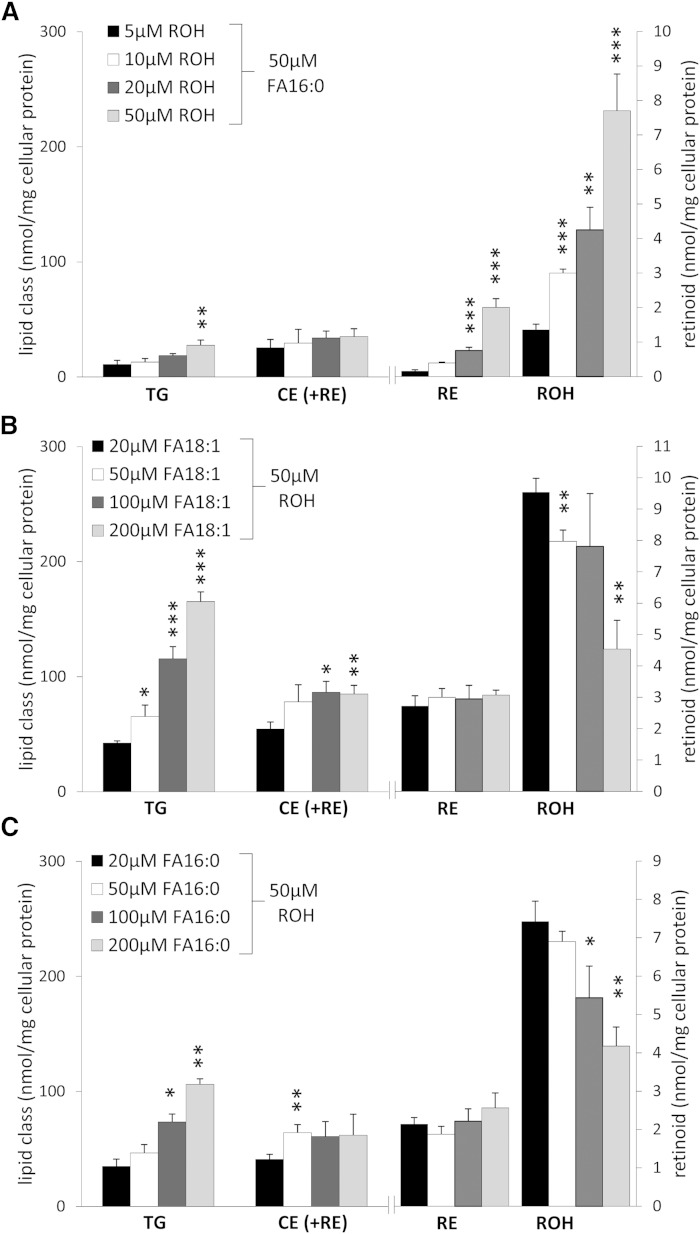
Lipid loading of HSC-T6 cells induces accumulation of neutral lipids. HSC-T6 cells were incubated for 16 h with increasing concentrations of ROH and a constant concentration of palmitic acid (FA16:0) (A), increasing concentrations of oleic acid (FA18:1) and a constant concentration of ROH (B), or increasing concentrations of FA16:0 and a constant concentration of ROH (C), as indicated. Cellular neutral lipids were extracted, quantified by HPLC-evaporative light scattering detection, and normalized to cell protein. CEs and REs coeluted and thus are labeled as CE (+RE). Data are presented as means + SD and are representative of three independent experiments (*P < 0.05, **P < 0.01, ***P < 0.001).

HSC-T6 is an established rat cell line which combines characteristics of quiescent (e.g., expression of retinoid-related proteins) and activated HSCs (e.g., fibroblast-like morphology) (24). For isolation of LDs, we loaded cells with ROH and FAs to promote LD formation and RE accumulation. First, we tested different loading conditions with increasing concentrations of ROH (5, 10, 20, and 50 μM) and a constant concentration of palmitic acid (FA16:0; 50 μM). Then, we measured cellular neutral lipid content (TGs, CEs, and REs) by HPLC. Although CEs could not be separately analyzed from REs because they coeluted, values reflected mostly CEs(+REs) because cellular CE content was much higher (∼10-fold) than that of REs. We initially chose FA16:0 for loading because the predominant naturally occurring RE species in the liver is retinyl-palmitate (RE16:0) (35). As expected, upon loading with increasing ROH concentrations, cellular ROH as well as RE content increased, while this was much less pronounced for cellular TG and CE(+RE) contents (**Fig. 1A**). Next, we examined whether increasing concentrations of oleic acid (FA18:1) or FA16:0 in combination with ROH (50 μM) would preferentially promote accumulation of any of the neutral lipid classes. In fact, we found that incubation of HSC-T6 cells with increasing FA18:1 (and constant ROH) concentration led to a dose-dependent increase in TG and CE(+RE) content, while for the latter, this increase was much less pronounced (Fig. 1B). Virtually no increase in RE content was observed, while cellular ROH content was actually decreased (Fig. 1B). Similarly, incubation of cells with increasing concentrations of FA16:0 led to an increase in cellular TGs, albeit to a lesser extent as compared with that of FA18:1-loaded cells (∼30%). Again, a minor increase in CE(+RE) content was observed, while RE content remained unchanged and ROH content again decreased (Fig. 1C). Taken together, the most pronounced increase in neutral lipid content was observed when cells were loaded with 200 μM FA18:1 + 50 μM ROH.

### Lipidomic profile of LDs of HSC-T6 cells

**Fig. 2.**
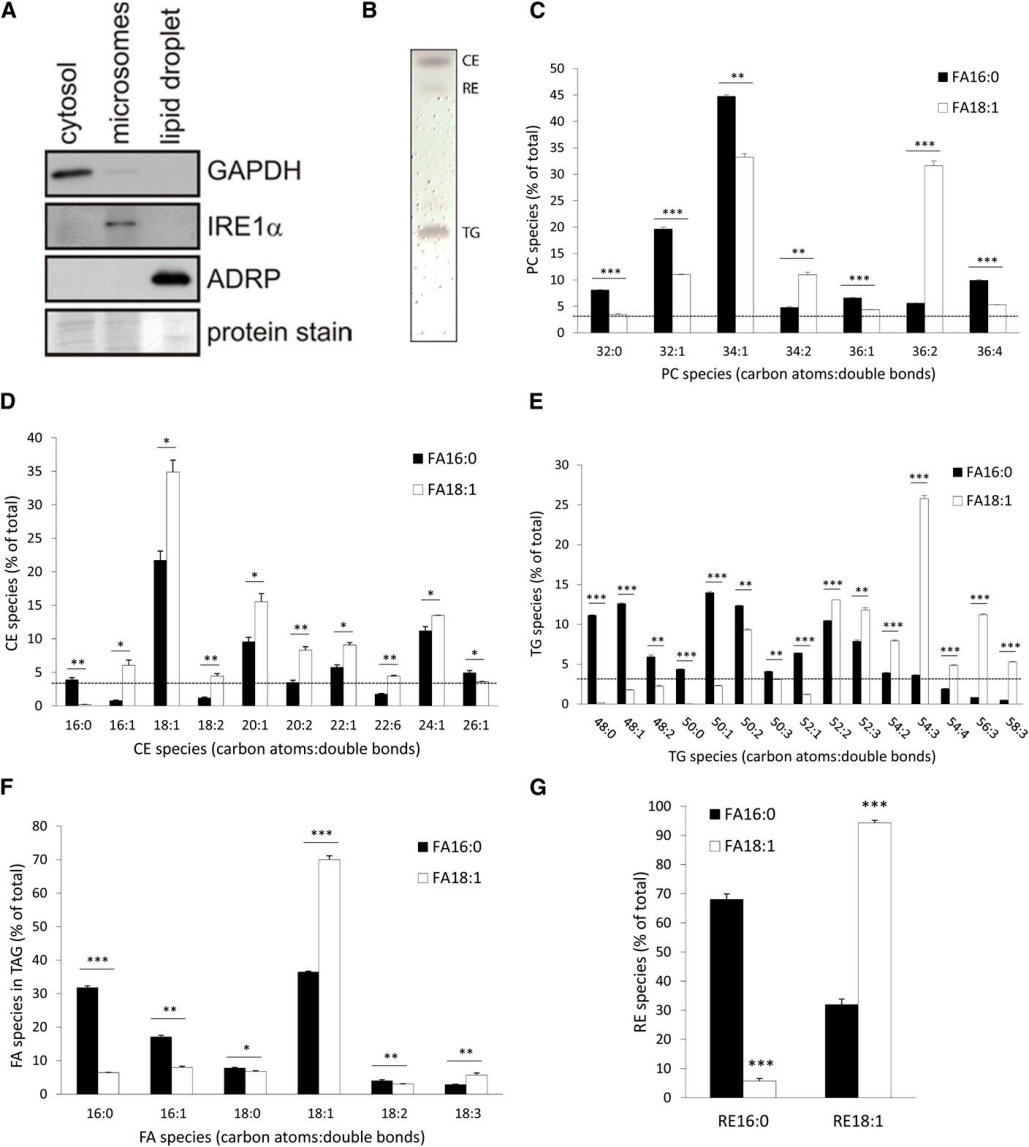
Lipidomic profile of LDs of HSC-T6 cells upon loading with ROH and palmitic or oleic acid. HSC-T6 cells were incubated with 50 μM ROH and 200 μM palmitic (FA16:0) or oleic acid (FA18:1) for 16 h (A–G). Proteins of isolated LDs and cell fractions were analyzed by Western blotting using marker proteins for cytosol (GAPDH), membrane (IRE1α), and the LD fraction (PLIN2/ADRP). Coomassie brilliant blue stain was used as loading control (A). Lipid extracts of isolated LDs were separated by TLC and lipids were visualized by sulfuric acid charring (B). The relative distribution of PC species (C), CE species (D); TG species (E), and FA species esterified to TGs (F) was analyzed by UPLC-qTOF. RE species (G) were analyzed by HPLC-fluorescence detection. Data are presented as means + SD and are representative of three independent experiments (*P < 0.05, **P < 0.01, ***P < 0.001).

For the analysis of the lipidomic profile, we loaded HSC-T6 cells with ROH+FA18:1 (50 μM and 200 μM, respectively) and isolated LDs by nitrogen-cavitation and ultracentrifugation. Assessment of the purity of LDs by Western blotting revealed that the LD fraction contained the LD marker protein, PLIN2 (PAT-protein family member, ADRP), while no signal was observed for GAPDH and IRE1α, marker proteins for the cytosol and the endoplasmic reticulum fraction, respectively (**Fig. 2A**). In contrast to the LD fraction, the cytosolic and membrane fractions were positive for GAPDH and IRE1α, respectively (Fig. 2A). To visualize the gross distribution of the major neutral lipids, we Folch-extracted LDs and separated lipids by TLC. As apparent from Fig. 2B, LDs contained comparable amounts of TGs and CEs, while the amount of REs was much less. This is in-line with the relative distribution of cellular neutral lipids (see Fig. 1A–C), where we found similar TG and CE contents, while that of RE was around 10-fold lower.

For the analysis of PC, TG, CE, and RE lipid species, LDs isolated from ROH+FA16:0- or ROH+FA18:1-loaded cells were subjected to UPLC-qTOF measurements. All lipid species with a relative abundance of >3% are depicted in Fig. 2C–G. The major PC species of LDs from ROH+FA16:0-loaded cells were PC32:0, PC32:1, PC34:1, and PC36:4, compatible with the idea that the PC species which are likely to contain one (PC32:1 and PC34:1) or two FA16:0s (PC32:0) are more prevalent (Fig. 2C). Loading of cells with ROH+FA18:1 shifted PC species of LDs toward FA18:1-containing species (e.g., PC34:2 and PC36:2 increased by ∼2- and 6-fold, respectively), while PC species likely to contain at least one FA16:0 (PC32:0, PC32:1, PC34:1) were reduced by around 10–15% (Fig. 2C). Most prominent changes in PC species upon FA18:1 versus FA16:0 loading were observed in PC36:2 (∼6-fold increase), suggesting that a significant portion of FA18:1 was incorporated into PC species without further elongation and desaturation.

Analysis of CE species of LD preparations of either FA16:0- or FA18:1-loaded cells revealed that irrespective of the FA species used for loading, the three most abundant CE species were CE18:1, CE20:1, and CE24:1 (Fig. 2D). Interestingly, LDs of FA16:0-loaded cells contained more CE16:0 and CE26:1 than those of FA18:1-loaded cells. In comparison, LDs of FA18:1-loaded cells contained more CE16:1, CE18:1, CE18:2, and, to a lesser degree, other unsaturated CE species (Fig. 2D). Taken together, CE18:1 was the major CE species in FA-loaded HSC-T6 cells, irrespective of whether FA16:0 or FA18:1 was used for FA loading of cells.

TGs are the most prominent storage pool for FAs. Because TGs contain three FAs esterified to the glycerol backbone, a large variety of combinations of different FA species is possible. For example, if three FA16:0s are esterified to the glycerol backbone, the resulting TG species has a carbon atom number of 48 with a desaturation (double bond) of zero, termed as TG48:0, while if three FA18:1s are esterified to the glycerol backbone, it gives a TG species termed as TG54:3. Analysis of TG species of LDs derived from ROH+FA16:0- or ROH+FA18:1-loaded HSC-T6 cells revealed that the distribution of TG species was very much dependent on the FA species used for lipid load­ing. LDs of FA16:0-loaded cells contained predominantly TG48:0, TG48:1, TG50:1, TG50:2, and TG52:2, with a relative abundance of >10%, all of which comprise TG species which contain at least one FA16:0 (Fig. 2E, black bars). In contrast, if cells were loaded with FA18:1, the TG species profile of LDs shifted toward TG species with increasing carbon numbers and desaturation. The most abundant TG species (>10% of total) were TG52:2, TG52:3, TG54:3, and TG56:3, again all of which contained at least one FA18:1 (Fig. 2E, white bars). MS/MS analysis of TG species revealed that upon FA16:0 loading of cells, around 30% of all FAs esterified to TG species were, in fact, FA16:0s and another ∼30% were FA18:1 (Fig. 2F). The relative abundance of other FA species (i.e., FA16:1, FA18:0, FA18:2, and FA18:3) was below 20%. In striking contrast, upon loading of cells with FA18:1, the utmost predominating FA species esterified to TGs was FA18:1 with a prevalence of ∼70% (Fig. 2F). Any of the other FA species determined accounted for less than 10%. These results clearly indicate that the majority of FAs taken up by HSC-T6 cells are immediately esterified to TGs and deposited within LDs.

In LD preparations of HSC-T6 cells, two RE species, namely RE16:0 and RE18:1, were detectable. Cells incubated with FA16:0 contained ∼70% RE16:0 and ∼30% RE18:1, whereas cells incubated with FA18:1 contained 10% RE16:0 and 90% RE18:1 (Fig. 2G). This indicates that both FA16:0 and FA18:1 are efficiently esterified to ROH.

### Proteomic analysis of LDs

**Fig. 3.**
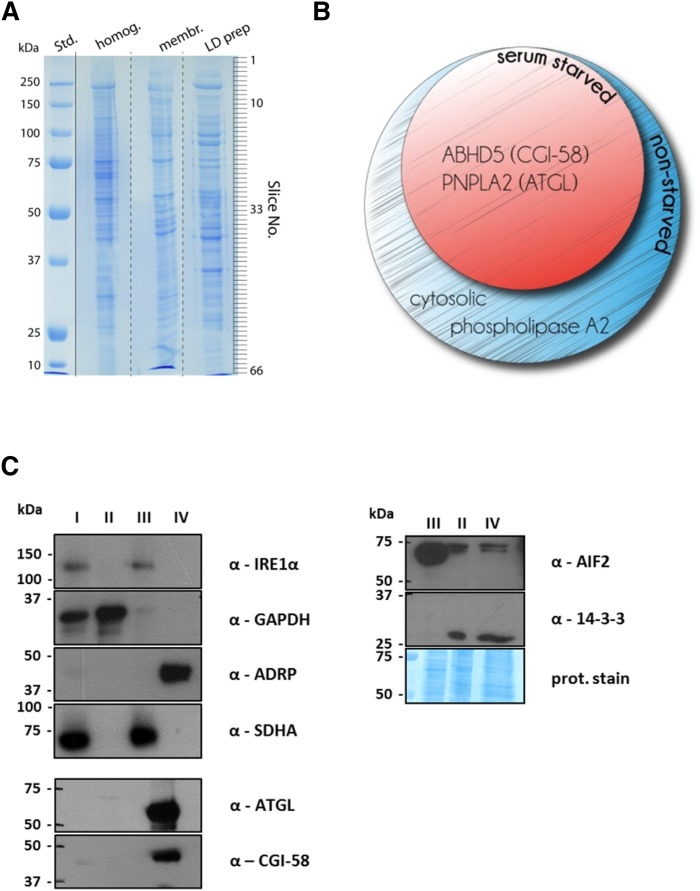
Proteomic profile of LDs derived from nonstarved or serum-starved HSC-T6 cells. HSC-T6 cells were incubated with 50 μM ROH and 200 μM oleic acid (FA18:1) for 16 h. In some cases, HSC-T6 cells were serum-starved for 2 h prior to cell harvest. Then, cytosolic, microsomal, and LD fractions were prepared by ultra-centrifugation. A: Proteins of various fractions were separated by SDS-PAGE and stained with Coomassie brilliant blue. The protein lane of the LD fraction was sliced (as indicated) and subjected to proteomic analysis. B: Venn diagram of α/β-hydrolase fold-containing proteins identified on LDs from nonstarved or serum-starved HSC-T6 cells. C: Proteins of the cell homogenate (I), the cytosol (II), the membrane/mitochondria (III), and the LD (IV) fraction were analyzed by Western blotting using antibodies against marker proteins GADPH for (II), IRE1α/SDHA for (III), and PLIN2/ADRP for (IV), as well as against selected antibodies (α-, as indicated). In some cases proteins on the Western blot membrane were stained with Coomassie brilliant blue.

For the proteomic analysis of LDs, we loaded cells with ROH+FA18:1 to promote LD formation. In some cases, cells were serum-starved prior to harvest. We reasoned that starvation might induce expression of neutral lipid-catabolizing enzymes. After isolation, LD proteins were separated by SDS-PAGE and analyzed by LC-MS/MS (**Fig. 3A**). As shown in Fig. 3A, the protein pattern of the LD fraction was distinct from that of the cell homogenate or the membrane fraction. In total, 168 and 131 LD-associated proteins were reproducibly identified in the nonstarved (supplementary Table 1) and serum-starved preparations (supplementary Table 2), respectively, using the constraints of at least five matching peptides and a protein score of >50. **Table 1** lists identified LD proteins which are directly or indirectly involved in the maintenance of lipid homeostasis. Not surprisingly, we not only found well-known LD-associated proteins such as ATGL, its coactivator protein CGI-58, and the PAT family member PLIN2/ADRP, but also found UBX domain containing protein 8 (UBXD8), apoptosis inducing factor 2 (AIF2), and various isoforms of tyrosine 3-monooxygenase/tryptophan 5-monooxygenase activation proteins (14-3-3/YWHA proteins). Because we aimed to identify neutral lipid hydrolyzing enzymes, we analyzed all identified proteins for the presence of an α/β-hydrolase fold and the active serine consensus motif GXSXG. We found three proteins, namely CGI-58, cytosolic phospholipase A2 α (cPLA2a), and ATGL, which contained an α/β-hydrolase fold, while two of them, cPLA2a and ATGL, also contained the GXSXG motif (**Table 2**). Interestingly, solely cPLA2a was found on LD preparations derived from nonstarved cells, while all of these α/β-hydrolase fold-containing proteins were found on LD preparations derived from serum-starved cells (Fig. 3B). To confirm purity of the LD preparation, we performed Western blotting analyses. Using marker proteins for cytosol (GAPDH) and the membrane and mitochondrial fraction [IRE1α and succinate dehydrogenase complex subunity A (SDHA), respectively], we observed no apparent contamination of the LD fraction (marker protein PLIN2/ADRP, see Fig. 3B). Signals for ATGL and CGI-58 were detectable in the LD fraction, which conformed to reports in the literature (36). In contrast, 14-3-3β and AIF2 were detected in the cytosol and LD fraction (Fig. 3C). Taken together, our proteomic analyses identified ATGL, CGI-58, and cPLA2a as LD-associated α/β-hydrolase fold-containing proteins in HSC-T6 cells.

**TABLE 1.** List of identified LD proteins which are involved in lipid metabolism

GI Number	Protein Name	LD Preparation	
		Nonstarved (Score/Peptides)	Serum-starved (Score/Peptides)
9507243	14-3-3 α/β (YWHAA/B)	185/5	160/11
13928824	14-3-3 *ε* (YWHAE)	290/13	192/11
9507245	14-3-3 ɣ (YWHAG)	192/5	147/9
1051270	14-3-3 ʐ (YWHAZ)	266/9	198/11
117558822	ABHD5 (CGI-58)	138/8	157/8
14192933	Aldehyde dehydrogenase, m.p.	326/19	—
6978501	Annexin A1	346/12	367/21
9845234	Annexin A2	360/10	349/19
55742832	Annexin A4	228/12	167/9
130502086	Annexin A6	407/18	—
213385255	Apoptosis-inducing factor 2 (AIF2)	—	262/13
126722674	Cytosolic phospholipase A2	63/7	—
51948390	Estradiol 17-β-dehydrogenase 11	—	270/15
62945246	FAS-associated factor 2 (UBXD8)	132/8	—
16923952	Long-chain FA-CoA ligase 3	243/15	460/27
16758426	Long-chain FA-CoA ligase 4	447/27	524/30
213688411	Lysophosphatidylcholine acyltransferase 1	89/6	115/6
13928780	NADPH-cytochrome P450 reductase	133/9	—
189095277	PNPLA2 (ATGL)	200/11	256/15
55742862	perilpin 2 (ADRP/PLIN2)	393/21	436/21
16758568	Phosphatidylinositol transfer protein β	162/12	—
11693142	Proliferating cell nuclear antigen	215/11	159/9
57164113	Sterol-4-α-carboxylate 3-dehydrogenase	146/9	—
17865351	Transitional endoplasmic reticulum ATPase	415/20	490/27
148747393	Trifunctional enzyme subunit α	205/11	—
GI, genInfo identifier; m.p., mitochondrial precursor.			

**TABLE 2.** List of identified LD proteins which contain an α/β-hydrolase fold and/or the active serine consensus motif G-X-S-X-G

GI Number	Protein Name	Motif	
		α/β-Hydrolase Fold	G-X-S-X-G
117558822	ABHD5 (CGI-58)	✓	—
126722674	Cytosolic phospholipase A2	✓	✓
189095277	PNPLA2 (ATGL)	✓	✓

A ✓ indicates proteins which contain the respective characteristic. GI, genInfo identifier.

### Neutral lipid hydrolytic activities of rATGL

**Fig. 4.**
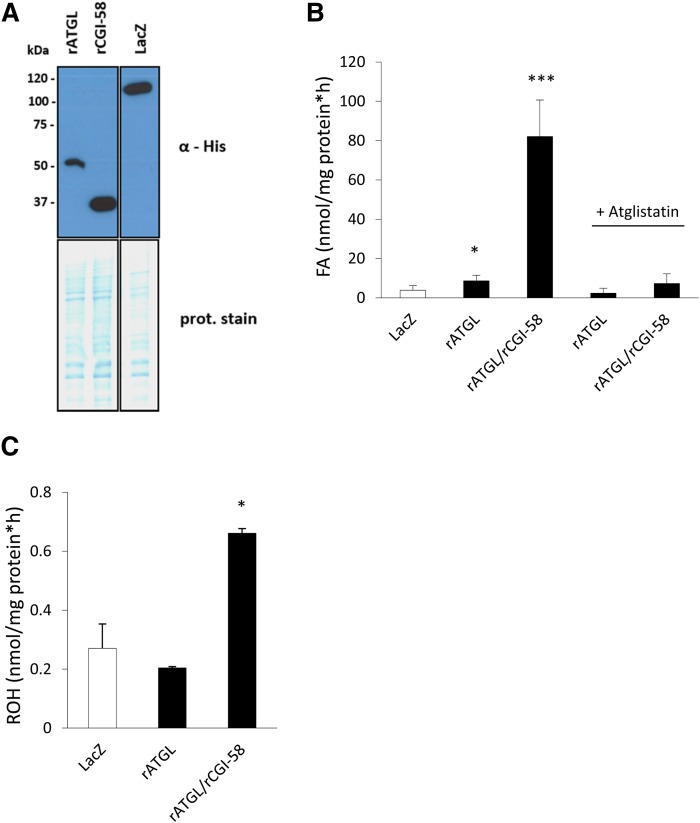
rATGL exhibits TG and RE hydrolase activity. COS-7 cells were transfected with plasmids encoding His-tagged rATGL, rat CGI-58, and LacZ as control. A: Expression of recombinant proteins was analyzed by Western blotting. Coomassie brilliant blue stain was used as loading control. B: Cell-lysates (40 μg protein) containing various recombinant proteins were incubated for 1 h with radiolabeled triolein and emulsified with PLs as substrate. In some cases Ai or solvent DMSO were added. After incubation, FAs were extracted and radioactivity determined by scintillation counting. C: Cell lysates (80 μg protein) were incubated for 1 h with retinyl-palmitate and emulsified with PLs as substrate. Then, retinyl-acetate was added as ISTD and retinoids were extracted. ROH content was determined by HPLC-fluorescence detection. All rates were calculated after blank-subtraction and were normalized to cell protein. Data are presented as means + SD and are representative of three independent experiments (*P < 0.05, ***P < 0.001).

To investigate whether the hydrolytic activity of rATGL against TGs and REs is similarly stimulated by the presence of rCGI-58 as compared with murine ATGL and CGI-58, we expressed recombinant His-tagged rat proteins in mammalian COS-7 cells. Western blotting analysis indicated expression of both recombinant proteins at the corresponding sizes (**Fig. 4A**). Then, lysates were subjected to in vitro neutral lipid hydrolase activity assays using triolein or retinyl-palmitate as substrates. Lysates containing rATGL exhibited ∼2-fold increased TG hydrolase activity as compared with LacZ-expressing control lysates (Fig. 4B). This activity was stimulated ∼10-fold by the addition of rCGI-58-containing lysates. Addition of the ATGL-specific inhibitor, Ai, completely blunted TG hydrolase activities of rATGL- and rATGL+rCGI-58-containing lysates to control levels (Fig. 4B). Furthermore, lysates containing rATGL+rCGI-58 exhibited ∼2.5-fold increased RE hydrolase activity as compared with LacZ control lysates (Fig. 4C). In summary, results indicate that rATGL, in the absence and presence of rCGI-58, hydrolyzes TGs, and that rATGL stimulated by rCGI-58 also hydrolyzes REs. In vitro activity of rATGL+rCGI-58 against TGs, however, was 100-fold higher as compared with RE hydrolase activity.

## DISCUSSION

HSCs were initially termed “fat-storing cells.” This derives from the large amount of neutral lipids which are stored in numerous LDs in the cytosol. These LDs contain not only TGs and CEs but also REs (17, 18). In fact, LDs of HSCs are the largest storage site for REs in the body (37, 38). These RE stores are critical for ensuring a constant supply of vitamin A. In times of nutritional vitamin A undersupply or deficiency, these RE stores are mobilized and eventually depleted (4, 39, 40). Furthermore, hepatic RE stores are also depleted upon alcoholic injury (13), a phenomenon which has attracted much attention. To date, the loss of the hepatic RE store is seen as a hallmark in the progression of liver disease from steatosis to fibrosis (8, 41–43). Despite this important role of the hepatic RE store under normo-physiological and pathological conditions, the identity of the protein(s) which facilitates the mobilization of neutral lipids (TGs, CEs, REs), in particular of REs, is still enigmatic.

In this study, we employed the rat HSC-T6 cell line as a source for the isolation of LDs. The aim was to determine the LD lipidome and proteome and to identify known or potential neutral lipid hydrolases by bioinformatic tools. We found that lipid species distribution of the TG and RE moiety was greatly affected by the FA species used for loading, while this was much less the case for CE and PC species. HSC-T6 cells accumulated about 10-fold more TGs and CEs than REs. Proteomic analysis revealed a number of well-known LD proteins, including ATGL, CGI-58, and PLIN2/ADRP. Interestingly, bioinformatic search for potential α/β-hydrolase fold proteins did not reveal additional hydrolases. In in vitro activity assays, we demonstrate that rATGL+rCGI-58 hydrolyze TGs and REs.

HSC-T6 cells do not contain sufficient amounts of LDs a priori. Thus, we loaded cells with ROH and FAs. In agreement with previous reports (24, 44), we found that loading of HSC-T6 cells with increasing concentrations of ROH and FAs triggered most prominently cellular retinoid and TG accumulation, respectively. Augmentation of RE stores of HSCs has also been demonstrated by feeding experiments in rats which, after receiving a low- or high-ROH diet, exhibited decreased or increased cellular retinoid content (>50-fold difference), respectively (17). Furthermore, in our HSC-T6 cell loading experiments, the highest relative retinoid content was observed upon loading of cells with ROH and FA16:0 (50 μM each), giving a relative distribution of 3:43:54 of RE:TG:CE, respectively. Several studies on isolated LDs of primary HSCs (17, 18) or sinusoidal cells (containing mostly HSCs) (45) reported higher RE than TG and total cholesterol contents, while in other studies the relative distribution of neutral lipids in primary HSCs or LDs of primary HSCs was reported to decrease in the order of TG>RE>CE (46, 47). These differences in the relative neutral lipid contents of HSCs are in line with our experimental data and results of vitamin A-diet studies in rats (17), and suggest that the neutral lipid content of HSCs is dynamic and responds to the nutritional availability of precursor lipids such as ROH and FAs. Interestingly, Testerink et al. (46) reported that upon activation of primary rat HSCs, the RE content was replaced by increasing TG and CE content. Because HSC-T6 cells exhibit an (partially) activated phenotype [fibroblast-like morphology, expression of α-SMA, collagenase-1 (48, 49)], changes in gene expression profile may contribute to the relative distribution of neutral lipid composition of their LDs. An explanation of the relatively low cellular RE, but relatively high ROH content, of HSC-T6 cells in our experiments could be that HSC-T6 cells did not efficiently take up and esterify extracellular ROH, which, after ethanolic injection into the culture media, was likely complexed to albumin (ROH:albumin) rather than to RBP4 (ROH:RBP4). Fortuna et al. (50) showed that HSCs do not take up ROH:albumin as efficiently as ROH:RBP4 (approximately two times less efficient). Furthermore, they found that, after incubation of HSCs with ROH:albumin, a large portion of ROH was actually associated with the membrane fraction, presumably not available for esterification (50).

Testerink et al. (46) compared the lipidomic profile of LDs derived from quiescent and activated primary rat HSCs. In that study, 15–20% of neutral lipids in quiescent HSCs consisted of REs, with the main RE species being RE16:0 (∼68% of all REs) and ∼9% being RE18:1, which is consistent with earlier reports (51, 52). No information on TG or PL species distributions of quiescent HSCs is given, except that TG species containing long-chain polyunsaturated FAs (i.e., FA20-22 with four or more double bonds) dramatically increased upon HSC activation. A lipidomic analysis of LDs derived from another hepatic cell type, murine primary hepatocytes, reported TG50:1–3, TG52:2–4, TG54:3–6, and TG56:6–8 as main species (53). In the same study, the major PC species were reported to be PC38:4 and PC38:6, accounting for ∼60% of all PC species; CE species were not analyzed. In comparison, we found in our lipidomic analysis that the RE and TG species distribution was very much dependent on the FA species used for loading of the cells. If cells were loaded with FA18:1, the main RE species was RE18:1 (∼90%) and FA18:1 was the predominating FA species in all TG species (∼70% of all FAs in the TG moiety). A similar shift in the FA distribution of RE and TG species was observed upon FA16:0 loading toward FA16:0-containing species. This responsiveness of the RE species distribution of HSC-T6 cells confirms observations reported in the initial characterization study of HSC-T6 cells (24). In contrast to the TG and PC species distribution of activated primary HSCs (46) and hepatocytes (53), in our lipidomic analysis of HSC-T6 cells, the major PC and TG species contained shorter FA chains with a lower degree of unsaturation (e.g., mostly TG48:0–1, TG50:1–3, and TG52:1–3; and PC32:1, PC34:1–2, and PC36:2). This suggests that the uptake of long-chain polyunsaturated FAs from the diet (such as FA18:2, FA18:3, or FA20:4) and/or the elongation and desaturation of FAs significantly contributes to the higher degree of very long-chain polyunsaturated FA species in TG as well as PL species contained in LDs of primary hepatocytes or activated HSCs.

The fact that the TG and RE species composition of HSC-T6 cells varied very much depending on the FA species used for loading indicates a flexibility which was not observed to that extent in the PC or CE pool. This flexibility suggests that the acyltransferases, diacylglycerol *O*-acyltransferases (DGATs), and lecithin:ROH *O*-acyltransferase (LRAT), which are mainly responsible for TG and RE biosynthesis, do not exert pronounced acyl donor preference. In fact, both DGAT1 and DGAT2 exhibit only an ∼2-fold preference for FA18:1 over FA16:0 (54). Similarly, LRAT exhibits virtually no preference for FA16:0 or FA18:1, although it exhibits a clear preference for short acyl chains (e.g., ∼50-fold higher activity for FA7:0) (55). Furthermore, this flexibility also indicates that the neutral lipid store of HSCs quickly responds to FA and ROH overload and channels excess lipids for storage. This view goes in line with the observation that the relative lipid composition of primary HSCs is, in fact, very much dependent on the lipid composition and vitamin A content of the diet (17).

Proteomic analyses of LDs identified a number of well-known LD proteins, including one member of the PAT protein family, lipid hydrolases, acyltransferases, and regulatory proteins. Interestingly, from the nine PAT protein family members (five classical perilipin family members, PLIN1–5), only PLIN2 (also known as adipophilin and ADRP) was found on the LD. In agreement with previous reports, PLIN2 is the most abundant PAT protein in the liver, while PLIN1 and PLIN5 are the most abundant PAT proteins in adipocytes and oxidative tissues, respectively (56–58). PLIN2 expression has also been shown to positively correlate with the degree of liver steatosis (58, 59). Consistent with these observations, the knockdown of PLIN2 in mice leads to reduced TG accumulation in the liver and reduced hepatic steatosis in response to high-fat feeding (60). Also, in primary HSCs, PLIN2 expression correlates with lipid content (61, 62), indicating that in parenchymal and nonparenchymal liver cells, PLIN2 is the dominating PAT protein. Interestingly, Straub et al. (58) also identified tail-interacting protein 47 (TIP47, annotated as PLIN3) on LDs of HSCs, as assessed by immunohistochemistry of steatotic human livers. In our proteomic analysis, however, we did not find TIP47, which might be due to a lower abundance of that protein. Because PLIN1 and PLIN5 are the solely established substrates for protein kinase A (63, 64), it can be concluded that the mobilization of neutral lipids in HSCs is not hormonally regulated. Interestingly, in human immortalized LX-2 HSCs, the upregulation of PLIN2 by FA+ROH loading was found to attenuate HSC activation, as evident by reduced expression of fibrogenic genes, while the opposite was found upon knockdown of PLIN2 expression by small interfering RNA (61). Furthermore, adenoviral overexpression of PLIN2 in rat primary HSCs promoted LD formation but could not reverse the activated phenotype of the cells, as assessed by collagen production (62). A very recent study demonstrates that levels of PLIN2 (and PLIN3) interfere with LD lipolysis and that chaperone-mediated degradation of PLIN2 (and PLIN3) promotes ATGL-mediated LD TG breakdown (65). Accordingly, it is feasible that lipid mobilization, including mobilization of REs, is determined by PLIN2 levels which may be determined by stress conditions.

On the LDs of HSC-T6 cells, we identified ATGL and CGI-58 (annotated as α/β-hydrolase domain containing 5), which are well-known to play an important role in lipid metabolism (in particular lipolysis) (66). Interestingly, we also found less established LD proteins with a role in lipolysis, such as 14-3-3epsilon (14-3-3e) and UBXD8 (annotated as FAS-associated factor 2). Notably, both have been shown to interact with ATGL and to affect its activity (67, 68). The presence of ATGL and interacting proteins (CGI-58, 14-3-3e, and UBXD8) suggests that HSC-T6 cells possess a lipolytic machinery for TG breakdown, which is mastered by ATGL. Furthermore, ATGL is known to be rate-limiting for TG catabolism and to also exhibit hydrolytic activity for REs (22, 69). Interestingly, rATGL activated by rCGI-58 (similar to murine ATGL) exhibited much higher hydrolytic activity for TGs than REs (22). Although ATGL has not been shown to be limiting for RE mobilization in primary murine HSCs (22), ATGL may still contribute to both TG and RE breakdown in HSCs.

We identified two other lipid modifying proteins, namely lysophosphatidylcholine acyltransferase 1 (LPCAT1) and cPLA2a. cPLA2a is known to specifically catalyze the hydrolysis of FAs (predominantly FA20:4) esterified at the *sn*-2 position of PLs (70). In contrast, LPCAT1 facilitates exactly the reverse reaction, by adding acyl-CoAs (preferentially FA16:0) to its acceptor 1-acyl-*sn*-glycero-3-phosphocholine (71). The presence of both LPCAT1 and cPLA2a suggests that LDs of HSC-T6 cells also contain PL modifying proteins which might be required for turnover of PL acyl-chains and/or for a specific remodeling of distinct PL species to conform, e.g., LD particle size or curvature of the PL layer.

The complete breakdown of TGs in adipocytes requires the sequential action of three enzymes: ATGL, hormone-sensitive lipase (HSL), and monoglyceride lipase (66). ATGL has been shown to be specific for the first step in lipolysis, to generate diglycerides. Because we identified neither HSL nor monoglyceride lipase on LDs of HSC-T6 cells, other enzymes could be required to facilitate the degradation of diglycerides and monoglycerides. It might be that additional hydrolytic enzymes were not identified because of very low abundance or that other, as yet unclassified, hydrolase enzymes exist, which exhibit hydrolytic activities for these reactions. Whether complete lipid breakdown is also under regulation of autophagy is currently not known.

It was surprising that we did not identify any known or potential RE hydrolases, such as members of the carboxyl-ester superfamily. Interestingly, we also did not identify PLRP2 or adiponutrin (PNPLA3) on the LD, because these proteins have been reported to be expressed in HSCs and to exhibit RE hydrolase activity (20, 21). One of the reasons could be that the expression levels of these proteins are relatively low, and thus were below the detection limit of our MS/MS analysis. Another possibility could be that some of the lipases on the LDs of HSCs are regulated by translocation from the cytosol to the LD, in a similar manner as shown for HSL (72). In case that the absence of ROH in the incubation media triggers the expression of a RE hydrolases, in addition to ATGL (in opposite to the expression of LRAT which is induced by retinoids), we should have found these proteins in our serum-starved data set. Furthermore, it is also possible that some of the LD-residing proteins got lost during the isolation procedure of LDs, and thus were not identified. From the identified lipid hydrolases, it is feasible that ATGL contributes to the hydrolysis of TGs and REs.

The proteomic analysis of LDs has the limitation that “false positive” proteins might be identified simply because of contamination with nonLD proteins. This may be caused by insufficient dilution of soluble proteins, but may also be caused by flotation of organelles or membrane fragments because they got trapped in the LD fraction. Our Western blotting analyses, however, showed no sign of contamination by other cellular fractions, ruling out apparent contamination. Because the LC-MS/MS method is not quantitative, we have no information on the abundance of any identified protein. Thus, it could be that some of the identified proteins are actually of rather low abundance (low-level contaminants) and were not detectable by Western blotting, but were identified by MS/MS. Furthermore, Western blotting analysis for ATGL, CGI-58, AIF2, and 14-3-3 confirmed that these proteins are in fact present in the LD preparation, confirming the validity of the MS/MS analysis.

In summary, this proteomic study identifies ATGL and CGI-58 as LD proteins of HSCs. Furthermore, we also demonstrate that the rat homolog of ATGL hydrolyzes TGs and that rATGL hydrolyzes REs in the presence of rCGI-58, and thus may participate in the breakdown of both neutral lipid esters in HSCs.

## Supplementary Material

Supplemental Tables
