# Supplementary material for: ATGL and CGI-58 are lipid droplet proteins of the hepatic stellate cell line HSC-T6
Source: J Lipid Res. 2015 Oct;56(10):1972–84. doi: 10.1194/jlr.M062372 (PMC4583087; doi:10.1194/jlr.M062372)
Supplement: Supplemental Tables [file supp_56_10_1972__index.html]

ATGL and CGI-58 are lipid droplet proteins of the hepatic stellate cell line HSC-T6 — Supplemental Tables 

# ATGL and CGI-58 are lipid droplet proteins of the hepatic stellate cell line HSC-T6

## Supplemental Data

Supplemental Tables 1 and 2

**Files in this Data Supplement:**

- Supplemental Table 1 - List of identified LD proteins derived from non-starved HSC-T6 cells
- Supplemental Table 2 - List of identified LD proteins derived from serum-starved HSC-T6 cells
