# Supplementary material for: ATGL and CGI-58 are lipid droplet proteins of the hepatic stellate cell line HSC-T6
Source: J Lipid Res. 2015 Oct;56(10):1972–84. doi: 10.1194/jlr.M062372 (PMC4583087; doi:10.1194/jlr.M062372)
Supplement: Supplemental Tables [file supp_M062372_Supplemental_Table_S1_non-starved.docx]

Table S1: List of identified LD proteins derived from non-starved HSC-T6 cells.

| **GI number** | **Protein name** | **SeqCoverage** | **Score** | **Peptides** |
| --- | --- | --- | --- | --- |
| 294506 | 14-3-3 protein [Rattus norvegicus] | 47,4 | 178,9 | 10 |
| 9507243 | 14-3-3 protein beta/alpha [Rattus norvegicus] | 22,8 | 185,4 | 5 |
| 13928824 | 14-3-3 protein epsilon [Rattus norvegicus] | 54,9 | 290,8 | 13 |
| 9507245 | 14-3-3 protein gamma [Rattus norvegicus] | 20,3 | 192,3 | 5 |
| 1051270 | 14-3-3 zeta isoform [Rattus norvegicus] | 40,4 | 266,1 | 9 |
| 8394221 | 40S ribosomal protein S3a [Rattus norvegicus] | 47,7 | 193,8 | 12 |
| 8393693 | 40S ribosomal protein SA [Rattus norvegicus] | 39,3 | 178,8 | 9 |
| 2541906 | 5-aminoimidazole-4-carboxamide ribonucleotide formyltransferase/IMP cyclohydrolase [Rattus norvegicus] | 23,2 | 206,1 | 11 |
| 11693176 | 60S acidic ribosomal protein P0 [Rattus norvegicus] | 32,5 | 169,3 | 9 |
| 25742763 | 78 kDa glucose-regulated protein precursor [Rattus norvegicus] | 49,1 | 554,5 | 30 |
| 91234898 | 84 kDa heat shock protein [Rattus norvegicus] | 45,5 | 604,1 | 36 |
| 117558822 | Abhd5 protein [Rattus norvegicus] | 33,1 | 138,4 | 8 |
| 40538860 | aconitate hydratase, mitochondrial precursor [Rattus norvegicus] | 42,2 | 449,4 | 27 |
| 16073616 | aldehyde dehydrogenase [Rattus norvegicus] | 53,7 | 298,9 | 18 |
| 14192933 | aldehyde dehydrogenase, mitochondrial precursor [Rattus norvegicus] | 46,1 | 326,0 | 19 |
| 202837 | aldolase A [Rattus norvegicus] | 46,7 | 274,1 | 14 |
| 62078615 | aldolase A-like 1 [Rattus norvegicus] | 22,0 | 179,9 | 9 |
| 6978491 | aldose reductase [Rattus norvegicus] | 18,7 | 93,6 | 6 |
| 27465603 | aldose reductase-related protein 2 [Rattus norvegicus] | 28,8 | 157,5 | 9 |
| 158186649 | alpha-enolase isoform 1 [Rattus norvegicus] | 61,8 | 391,6 | 22 |
| 6978501 | annexin A1 [Rattus norvegicus] | 36,7 | 346,0 | 12 |
| 9845234 | annexin A2 [Rattus norvegicus] | 29,8 | 360,1 | 10 |
| 55742832 | annexin A4 [Rattus norvegicus] | 45,5 | 228,1 | 12 |
| 130502086 | annexin A6 [Rattus norvegicus] | 26,2 | 407,2 | 18 |
| 213385255 | apoptosis-inducing factor, mitochondrion-associated 2 [Rattus norvegicus] | 31,1 | 255,5 | 14 |
| 1374715 | ATP synthase beta subunit [Rattus norvegicus] | 71,6 | 417,1 | 21 |
| 126723393 | beta-enolase [Rattus norvegicus] | 25,6 | 109,1 | 8 |
| 48675845 | bifunctional purine biosynthesis protein PURH [Rattus norvegicus] | 26,0 | 222,3 | 12 |
| 488838 | CaBP1 [Rattus norvegicus] | 35,3 | 188,5 | 11 |
| 25282419 | calnexin precursor [Rattus norvegicus] | 15,6 | 218,7 | 8 |
| 11693172 | calreticulin precursor [Rattus norvegicus] | 52,7 | 318,3 | 22 |
| 1778213 | chaperonin 60 [Rattus norvegicus] | 55,5 | 449,8 | 28 |
| 149059759 | chaperonin subunit 8 (theta) (predicted), isoform CRA_a [Rattus norvegicus] | 44,2 | 381,9 | 21 |
| 203734 | cytokeratin 8 polypeptide [Rattus norvegicus] | 36,2 | 318,3 | 20 |
| 58219062 | cytosolic non-specific dipeptidase [Rattus norvegicus] | 34,5 | 214,7 | 13 |
| 1143305 | cytosolic phospholipase A2 [Rattus norvegicus] | 9,7 | 62,8 | 7 |
| 13928850 | D-3-phosphoglycerate dehydrogenase [Rattus norvegicus] | 30,2 | 222,5 | 12 |
| 11968118 | desmin [Rattus norvegicus] | 6,8 | 73,3 | 5 |
| 40786469 | dihydrolipoyl dehydrogenase, mitochondrial precursor [Rattus norvegicus] | 22,2 | 146,0 | 9 |
| 6981486 | dolichyl-diphosphooligosaccharide--protein glycosyltransferase subunit 1 [Rattus norvegicus] | 27,6 | 236,0 | 14 |
| 204070 | electron transfer flavoprotein alpha-subunit [Rattus norvegicus] | 25,7 | 84,6 | 6 |
| 57527204 | electron transfer flavoprotein subunit alpha, mitochondrial precursor [Rattus norvegicus] | 31,8 | 104,8 | 8 |
| 28460696 | elongation factor 1-alpha 1 [Rattus norvegicus] | 25,1 | 330,4 | 11 |
| 8393296 | elongation factor 2 [Rattus norvegicus] | 41,2 | 624,3 | 35 |
| 210032365 | endoplasmin precursor [Rattus norvegicus] | 34,7 | 612,9 | 28 |
| 59808815 | Enolase 1, (alpha) [Rattus norvegicus] | 52,1 | 356,7 | 20 |
| 51948390 | estradiol 17-beta-dehydrogenase 11 [Rattus norvegicus] | 45,3 | 276,6 | 12 |
| 56605748 | eukaryotic initiation factor 4A-II [Rattus norvegicus] | 29,0 | 183,9 | 10 |
| 52138521 | ezrin [Rattus norvegicus] | 39,9 | 426,8 | 27 |
| 203033 | F1-ATPase beta subunit [Rattus norvegicus] | 71,2 | 269,4 | 15 |
| 62945246 | FAS-associated factor 2 [Rattus norvegicus] | 36,7 | 132,0 | 8 |
| 4838513 | foocen-m2 [Rattus norvegicus] | 18,0 | 91,7 | 5 |
| 6978487 | fructose-bisphosphate aldolase A [Rattus norvegicus] | 52,2 | 297,9 | 15 |
| 984553 | G protein beta 1 subunit [Rattus norvegicus] | 15,0 | 86,0 | 5 |
| 26023949 | gamma-enolase [Rattus norvegicus] | 21,2 | 107,3 | 7 |
| 157818781 | glucosidase 2 subunit beta [Rattus norvegicus] | 20,0 | 144,0 | 13 |
| 8393418 | glyceraldehyde-3-phosphate dehydrogenase [Rattus norvegicus] | 55,0 | 275,3 | 14 |
| 15100041 | G-protein beta-2 subunit [Rattus norvegicus] | 23,6 | 84,5 | 5 |
| 262272076 | GTP-binding nuclear protein Ran, testis-specific isoform [Rattus norvegicus] | 24,5 | 115,3 | 5 |
| 71089939 | guanine nucleotide binding protein beta 2 [Rattus norvegicus] | 29,2 | 139,5 | 8 |
| 18543331 | guanine nucleotide-binding protein subunit beta-2-like 1 [Rattus norvegicus] | 41,0 | 283,0 | 12 |
| 13242237 | heat shock cognate 71 kDa protein [Rattus norvegicus] | 52,8 | 596,8 | 29 |
| 56383 | heat shock protein (hsp60) precursor [Rattus norvegicus] | 59,9 | 464,3 | 29 |
| 51859516 | Heat shock protein 90kDa alpha (cytosolic), class B member 1 [Rattus norvegicus] | 45,5 | 611,6 | 36 |
| 94400790 | heat shock protein beta-1 [Rattus norvegicus] | 52,7 | 186,4 | 10 |
| 28467005 | heat shock protein HSP 90-alpha [Rattus norvegicus] | 41,2 | 550,3 | 32 |
| 11177910 | heat shock-related 70 kDa protein 2 [Rattus norvegicus] | 14,2 | 220,1 | 9 |
| 13592093 | hsc70-interacting protein [Rattus norvegicus] | 25,8 | 172,1 | 10 |
| 77404375 | hypoxia up-regulated protein 1 [Rattus norvegicus] | 29,0 | 385,0 | 28 |
| 202549 | iodothyronine 5' monodeiodinase [Rattus norvegicus] | 47,9 | 353,6 | 24 |
| 57012436 | keratin, type I cytoskeletal 10 [Rattus norvegicus] | 23,4 | 260,2 | 15 |
| 51591909 | keratin, type I cytoskeletal 13 [Rattus norvegicus] | 8,0 | 102,8 | 5 |
| 56912233 | keratin, type I cytoskeletal 14 [Rattus norvegicus] | 8,5 | 226,0 | 5 |
| 51591903 | keratin, type I cytoskeletal 15 [Rattus norvegicus] | 7,6 | 121,3 | 5 |
| 56847618 | keratin, type I cytoskeletal 16 [Rattus norvegicus] | 10,2 | 84,1 | 5 |
| 47087085 | keratin, type I cytoskeletal 17 [Rattus norvegicus] | 12,5 | 163,2 | 7 |
| 42409519 | keratin, type I cytoskeletal 19 [Rattus norvegicus] | 13,7 | 95,4 | 8 |
| 57012368 | keratin, type II cytoskeletal 1b [Rattus norvegicus] | 9,1 | 125,6 | 7 |
| 57114290 | keratin, type II cytoskeletal 2 epidermal [Rattus norvegicus] | 9,2 | 119,4 | 9 |
| 57012372 | keratin, type II cytoskeletal 2 oral [Rattus norvegicus] | 5,0 | 71,3 | 5 |
| 57012360 | keratin, type II cytoskeletal 4 [Rattus norvegicus] | 8,0 | 122,7 | 7 |
| 50233797 | keratin, type II cytoskeletal 5 [Rattus norvegicus] | 15,5 | 267,4 | 11 |
| 155369696 | keratin, type II cytoskeletal 6A [Rattus norvegicus] | 12,1 | 171,9 | 8 |
| 40786432 | keratin, type II cytoskeletal 8 [Rattus norvegicus] | 36,4 | 318,3 | 21 |
| 114145409 | keratin, type II cytoskeletal cochleal [Rattus norvegicus] | 8,7 | 109,9 | 7 |
| 56788780 | Krt19 protein [Rattus norvegicus] | 13,0 | 95,4 | 8 |
| 453180 | lamin A [Rattus norvegicus] | 28,0 | 396,4 | 18 |
| 16758782 | lamin-B1 [Rattus norvegicus] | 38,7 | 467,6 | 27 |
| 38014570 | Ldha protein [Rattus norvegicus] | 61,9 | 300,8 | 17 |
| 56605670 | leucine-rich repeat-containing protein 59 [Rattus norvegicus] | 20,9 | 107,9 | 6 |
| 16923952 | long-chain-fatty-acid--CoA ligase 3 [Rattus norvegicus] | 24,2 | 243,1 | 15 |
| 16758426 | long-chain-fatty-acid--CoA ligase 4 [Rattus norvegicus] | 47,0 | 446,6 | 27 |
| 103485105 | LPC acyltransferase [Rattus norvegicus] | 10,4 | 71,4 | 5 |
| 37361912 | LRRGT00113 [Rattus norvegicus] | 33,5 | 220,4 | 15 |
| 213688411 | lysophosphatidylcholine acyltransferase 1 [Rattus norvegicus] | 11,1 | 88,6 | 6 |
| 206205 | M2 pyruvate kinase [Rattus norvegicus] | 62,0 | 497,2 | 28 |
| 42476181 | malate dehydrogenase, mitochondrial precursor [Rattus norvegicus] | 54,7 | 279,4 | 15 |
| 82654202 | methyltransferase-like protein 7A [Rattus norvegicus] | 32,0 | 179,7 | 7 |
| 10637996 | mitochondrial aconitase [Rattus norvegicus] | 39,0 | 408,0 | 25 |
| 25990263 | mitochondrial aldehyde dehydrogenase [Rattus norvegicus] | 48,8 | 298,9 | 18 |
| 45737868 | mitochondrial aldehyde dehydrogenase precursor [Rattus norvegicus] | 43,3 | 276,6 | 17 |
| 89574117 | mitochondrial malate dehydrogenase 2, NAD [Rattus norvegicus] | 60,8 | 238,2 | 14 |
| 13540689 | moesin [Rattus norvegicus] | 33,6 | 446,1 | 24 |
| 13928704 | myosin-10 [Rattus norvegicus] | 15,5 | 423,4 | 28 |
| 282158051 | myosin-11 [Rattus norvegicus] | 7,8 | 297,0 | 16 |
| 162287127 | myosin-14 [Rattus norvegicus] | 4,1 | 166,1 | 9 |
| 6981236 | myosin-9 [Rattus norvegicus] | 37,2 | 1186,0 | 74 |
| 205660 | NADPH-cytochrome P-450 oxidoreductase [Rattus norvegicus] | 12,8 | 133,0 | 9 |
| 5410338 | NADPH-dependent thioredoxin reductase [Rattus norvegicus] | 12,1 | 88,0 | 6 |
| 6822249 | Nogo-B protein [Rattus norvegicus] | 18,9 | 91,7 | 5 |
| 189095277 | patatin-like phospholipase domain-containing protein 2 [Rattus norvegicus] | 26,2 | 200,4 | 11 |
| 55742862 | perilipin-2 [Rattus norvegicus] | 63,0 | 393,4 | 21 |
| 72679690 | Peripherin [Rattus norvegicus] | 9,9 | 71,3 | 5 |
| 16758568 | phosphatidylinositol transfer protein beta isoform [Rattus norvegicus] | 40,6 | 162,1 | 12 |
| 40254752 | phosphoglycerate kinase 1 [Rattus norvegicus] | 57,6 | 387,3 | 21 |
| 58865830 | phosphoglycerate kinase 2 [Rattus norvegicus] | 13,2 | 87,1 | 5 |
| 57164095 | pirin [Rattus norvegicus] | 39,9 | 199,5 | 12 |
| 38181543 | Pkm2 protein [Rattus norvegicus] | 60,6 | 445,1 | 25 |
| 157817831 | poly(A) binding protein, cytoplasmic 2 [Rattus norvegicus] | 13,8 | 113,4 | 9 |
| 157818977 | poly(A) binding protein, cytoplasmic 3 [Rattus norvegicus] | 18,5 | 193,0 | 13 |
| 19705459 | polyadenylate-binding protein 1 [Rattus norvegicus] | 29,4 | 286,7 | 20 |
| 62078699 | probable saccharopine dehydrogenase [Rattus norvegicus] | 36,8 | 224,7 | 12 |
| 11693142 | proliferating cell nuclear antigen [Rattus norvegicus] | 50,2 | 214,7 | 11 |
| 51948384 | proliferation-associated protein 2G4 [Rattus norvegicus] | 43,2 | 249,0 | 15 |
| 51036657 | prolyl 4-hydroxylase subunit alpha-1 precursor [Rattus norvegicus] | 24,4 | 180,2 | 11 |
| 6981324 | protein disulfide-isomerase [Rattus norvegicus] | 45,5 | 362,0 | 24 |
| 8393322 | protein disulfide-isomerase A3 precursor [Rattus norvegicus] | 52,7 | 447,7 | 25 |
| 16758712 | protein disulfide-isomerase A4 precursor [Rattus norvegicus] | 35,3 | 361,0 | 24 |
| 52345385 | protein disulfide-isomerase A6 [Rattus norvegicus] | 34,2 | 188,5 | 11 |
| 16757994 | pyruvate kinase isozymes M1/M2 [Rattus norvegicus] | 52,7 | 439,8 | 25 |
| 40254781 | rab GDP dissociation inhibitor beta [Rattus norvegicus] | 13,3 | 267,8 | 6 |
| 56799432 | radixin [Rattus norvegicus] | 23,2 | 213,6 | 16 |
| 149052054 | rCG34378, isoform CRA_h [Rattus norvegicus] | 28,1 | 169,3 | 7 |
| 149064296 | rCG46767, isoform CRA_c [Rattus norvegicus] | 40,7 | 485,6 | 28 |
| 157819753 | reticulocalbin-1 [Rattus norvegicus] | 13,9 | 58,7 | 5 |
| 38512106 | Ribophorin I [Rattus norvegicus] | 28,9 | 249,8 | 15 |
| 2920827 | ribosomal protein S2 [Rattus norvegicus] | 28,1 | 154,3 | 7 |
| 56605722 | serine hydroxymethyltransferase, mitochondrial [Rattus norvegicus] | 52,4 | 362,9 | 20 |
| 8393057 | serpin H1 precursor [Rattus norvegicus] | 49,9 | 362,3 | 18 |
| 55824765 | Serpinh1 protein [Rattus norvegicus] | 50,4 | 378,5 | 18 |
| 148747124 | short-chain dehydrogenase/reductase 3 [Rattus norvegicus] | 42,1 | 197,0 | 10 |
| 57164113 | sterol-4-alpha-carboxylate 3-dehydrogenase, decarboxylating [Rattus norvegicus] | 24,6 | 146,3 | 9 |
| 154816168 | stress-70 protein, mitochondrial [Rattus norvegicus] | 44,0 | 448,8 | 25 |
| 20302113 | stress-induced-phosphoprotein 1 [Rattus norvegicus] | 40,0 | 511,3 | 23 |
| 76096306 | synaptic vesicle membrane protein VAT-1 homolog [Rattus norvegicus] | 58,2 | 309,3 | 16 |
| 51890219 | T-complex protein 1 subunit epsilon [Rattus norvegicus] | 35,3 | 296,5 | 20 |
| 157819651 | T-complex protein 1 subunit eta [Rattus norvegicus] | 34,0 | 292,5 | 18 |
| 40018616 | T-complex protein 1 subunit gamma [Rattus norvegicus] | 42,8 | 399,9 | 22 |
| 76253725 | T-complex protein 1 subunit zeta [Rattus norvegicus] | 29,8 | 257,0 | 15 |
| 78191795 | thioredoxin reductase 1, cytoplasmic [Rattus norvegicus] | 18,0 | 123,3 | 8 |
| 51858886 | Tra1 protein [Rattus norvegicus] | 42,2 | 402,0 | 26 |
| 281306746 | transferrin receptor protein 1 [Rattus norvegicus] | 8,4 | 54,1 | 6 |
| 17865351 | transitional endoplasmic reticulum ATPase [Rattus norvegicus] | 26,4 | 415,0 | 20 |
| 12018252 | transketolase [Rattus norvegicus] | 26,0 | 263,6 | 15 |
| 148747393 | trifunctional enzyme subunit alpha, mitochondrial precursor [Rattus norvegicus] | 15,7 | 205,3 | 11 |
| 6981672 | tropomyosin alpha-4 chain [Rattus norvegicus] | 29,0 | 158,1 | 9 |
| 58865558 | tubulin alpha-1C chain [Rattus norvegicus] | 56,8 | 298,3 | 17 |
| 66730465 | tubulin alpha-8 chain [Rattus norvegicus] | 30,3 | 181,0 | 9 |
| 38328248 | Tubulin, alpha 1A [Rattus norvegicus] | 61,9 | 318,4 | 18 |
| 38454226 | tumor protein D54 [Rattus norvegicus] | 51,8 | 166,9 | 11 |
| 55250051 | Txnrd1 protein [Rattus norvegicus] | 15,6 | 123,3 | 8 |
| 71043618 | tyrosyl-tRNA synthetase, cytoplasmic [Rattus norvegicus] | 35,8 | 298,2 | 20 |
| 38051979 | Vdac1 protein [Rattus norvegicus] | 58,3 | 198,9 | 12 |
| 14389299 | vimentin [Rattus norvegicus] | 51,5 | 473,3 | 26 |
| 149031250 | vinculin (predicted), isoform CRA_a [Rattus norvegicus] | 7,3 | 642,1 | 8 |
| 4105605 | voltage dependent anion channel [Rattus norvegicus] | 51,2 | 168,0 | 11 |
| 13786202 | voltage-dependent anion-selective channel protein 2 [Rattus norvegicus] | 30,5 | 117,5 | 7 |
| 157819953 | V-type proton ATPase catalytic subunit A [Rattus norvegicus] | 15,7 | 99,7 | 9 |
|  |  |  |  |  |
