# Supplementary material for: ATGL and CGI-58 are lipid droplet proteins of the hepatic stellate cell line HSC-T6
Source: J Lipid Res. 2015 Oct;56(10):1972–84. doi: 10.1194/jlr.M062372 (PMC4583087; doi:10.1194/jlr.M062372)
Supplement: Supplemental Tables [file supp_M062372_Supplemental_Table_S2_serum-starved-1.docx]

Table S2: List of identified LD proteins derived from serum-starved HSC-T6 cells.

| **GI number** | **Protein name** | **Coverage** | **Score** | **Peptides** |
| --- | --- | --- | --- | --- |
| 294506 | 14-3-3 protein [Rattus norvegicus] | 42,0 | 144,1 | 8 |
| 9507243 | 14-3-3 protein beta/alpha [Rattus norvegicus] | 42,7 | 160,3 | 11 |
| 13928824 | 14-3-3 protein epsilon [Rattus norvegicus] | 49,0 | 192,3 | 11 |
| 9507245 | 14-3-3 protein gamma [Rattus norvegicus] | 37,3 | 146,5 | 9 |
| 1051270 | 14-3-3 zeta isoform [Rattus norvegicus] | 47,8 | 197,9 | 11 |
| 639865 | 2,3-oxidosqualene:lanosterol cyclase [Rattus norvegicus] | 51,0 | 522,7 | 29 |
| 8393693 | 40S ribosomal protein SA [Rattus norvegicus] | 58,3 | 256,8 | 13 |
| 25742763 | 78 kDa glucose-regulated protein precursor [Rattus norvegicus] | 45,9 | 421,4 | 26 |
| 91234898 | 84 kDa heat shock protein [Rattus norvegicus] | 50,3 | 603,5 | 35 |
| 117558822 | Abhd5 protein [Rattus norvegicus] | 35,5 | 156,5 | 8 |
| 32527749 | Ac2-233 [Rattus norvegicus] | 15,5 | 275,5 | 12 |
| 6978499 | acidic leucine-rich nuclear phosphoprotein 32 family member A [Rattus norvegicus] | 22,7 | 86,4 | 7 |
| 18777770 | acidic leucine-rich nuclear phosphoprotein 32 family member B [Rattus norvegicus] | 30,2 | 136,7 | 8 |
| 49256643 | Actn1 protein [Rattus norvegicus] | 37,2 | 454,5 | 28 |
| 209862801 | alanyl-tRNA synthetase, cytoplasmic [Rattus norvegicus] | 43,2 | 478,2 | 28 |
| 13591894 | alcohol dehydrogenase [NADP+] [Rattus norvegicus] | 39,1 | 207,3 | 11 |
| 202837 | aldolase A [Rattus norvegicus] | 56,6 | 310,5 | 16 |
| 62078615 | aldolase A-like 1 [Rattus norvegicus] | 29,4 | 193,8 | 10 |
| 6636119 | alpha-actinin 4 [Rattus norvegicus] | 33,6 | 372,4 | 24 |
| 158186649 | alpha-enolase isoform 1 [Rattus norvegicus] | 71,2 | 428,1 | 24 |
| 6978501 | annexin A1 [Rattus norvegicus] | 63,0 | 366,6 | 21 |
| 9845234 | annexin A2 [Rattus norvegicus] | 52,2 | 348,9 | 19 |
| 6978503 | annexin A3 [Rattus norvegicus] | 55,6 | 297,2 | 18 |
| 55742832 | annexin A4 [Rattus norvegicus] | 36,1 | 167,0 | 9 |
| 6978505 | annexin A5 [Rattus norvegicus] | 57,4 | 277,1 | 16 |
| 213385255 | apoptosis-inducing factor, mitochondrion-associated 2 [Rattus norvegicus] | 37,5 | 261,9 | 13 |
| 6980970 | aspartate aminotransferase, cytoplasmic [Rattus norvegicus] | 39,2 | 224,3 | 13 |
| 126723393 | beta-enolase [Rattus norvegicus] | 16,1 | 57,2 | 5 |
| 38018016 | brain-specific alpha actinin 1 isoform [Rattus norvegicus] | 37,4 | 472,1 | 29 |
| 488838 | CaBP1 [Rattus norvegicus] | 31,8 | 153,2 | 9 |
| 11693172 | calreticulin precursor [Rattus norvegicus] | 46,6 | 271,3 | 17 |
| 76779821 | Carbonyl reductase 1 [Rattus norvegicus] | 39,4 | 167,8 | 10 |
| 9506475 | cell division protein kinase 1 [Rattus norvegicus] | 19,9 | 87,1 | 5 |
| 149059759 | chaperonin subunit 8 (theta) (predicted), isoform CRA_a [Rattus norvegicus] | 34,1 | 283,0 | 15 |
| 50657380 | chloride intracellular channel protein 1 [Rattus norvegicus] | 31,5 | 125,9 | 7 |
| 13929166 | chloride intracellular channel protein 4 [Rattus norvegicus] | 27,3 | 90,6 | 6 |
| 220684 | cytosolic aspartate aminotransferase [Rattus norvegicus] | 47,7 | 275,3 | 16 |
| 13928850 | D-3-phosphoglycerate dehydrogenase [Rattus norvegicus] | 42,6 | 297,5 | 16 |
| 56090381 | dehydrogenase/reductase SDR family member 1 [Rattus norvegicus] | 86,3 | 421,9 | 22 |
| 157824028 | ELMO domain-containing protein 2 [Rattus norvegicus] | 28,0 | 101,4 | 8 |
| 51948418 | elongation factor 1-gamma [Rattus norvegicus] | 46,0 | 296,5 | 16 |
| 8393296 | elongation factor 2 [Rattus norvegicus] | 57,8 | 769,3 | 42 |
| 210032365 | endoplasmin precursor [Rattus norvegicus] | 28,9 | 390,3 | 22 |
| 38649320 | Eno1 protein [Rattus norvegicus] | 59,1 | 397,5 | 20 |
| 59808815 | Enolase 1, (alpha) [Rattus norvegicus] | 54,6 | 353,5 | 18 |
| 51948390 | estradiol 17-beta-dehydrogenase 11 [Rattus norvegicus] | 51,0 | 270,2 | 15 |
| 56605748 | eukaryotic initiation factor 4A-II [Rattus norvegicus] | 34,4 | 204,8 | 12 |
| 52138521 | ezrin [Rattus norvegicus] | 19,3 | 137,4 | 12 |
| 22324680 | FK506 binding protein 4 [Rattus norvegicus] | 32,1 | 195,8 | 11 |
| 6978487 | fructose-bisphosphate aldolase A [Rattus norvegicus] | 62,1 | 332,8 | 17 |
| 56605662 | galactokinase [Rattus norvegicus] | 34,4 | 192,3 | 11 |
| 25453420 | glutathione S-transferase P [Rattus norvegicus] | 50,0 | 136,3 | 8 |
| 56188 | glyceraldehyde 3-phosphate-dehydrogenase [Rattus norvegicus] | 68,8 | 360,1 | 19 |
| 18543331 | guanine nucleotide-binding protein subunit beta-2-like 1 [Rattus norvegicus] | 64,4 | 258,5 | 15 |
| 13242237 | heat shock cognate 71 kDa protein [Rattus norvegicus] | 57,0 | 544,6 | 28 |
| 56383 | heat shock protein (hsp60) precursor [Rattus norvegicus] | 42,4 | 314,3 | 18 |
| 58865372 | heat shock protein 105 kDa [Rattus norvegicus] | 31,6 | 368,5 | 21 |
| 51859516 | Heat shock protein 90kDa alpha (cytosolic), class B member 1 [Rattus norvegicus] | 54,8 | 610,6 | 37 |
| 94400790 | heat shock protein beta-1 [Rattus norvegicus] | 54,2 | 199,8 | 12 |
| 13592093 | hsc70-interacting protein [Rattus norvegicus] | 13,1 | 75,3 | 5 |
| 1906812 | inducible carbonyl reductase [Rattus norvegicus] | 35,4 | 149,3 | 9 |
| 71534283 | interferon-induced GTP-binding protein Mx2 [Rattus norvegicus] | 31,1 | 283,2 | 17 |
| 202549 | iodothyronine 5' monodeiodinase [Rattus norvegicus] | 57,9 | 390,5 | 25 |
| 2443314 | keratin 14 [Rattus norvegicus] | 20,4 | 51,4 | 5 |
| 57012436 | keratin, type I cytoskeletal 10 [Rattus norvegicus] | 19,6 | 194,8 | 14 |
| 56847618 | keratin, type I cytoskeletal 16 [Rattus norvegicus] | 11,2 | 55,7 | 5 |
| 47087085 | keratin, type I cytoskeletal 17 [Rattus norvegicus] | 25,0 | 202,6 | 13 |
| 50233797 | keratin, type II cytoskeletal 5 [Rattus norvegicus] | 10,3 | 131,7 | 6 |
| 155369696 | keratin, type II cytoskeletal 6A [Rattus norvegicus] | 8,7 | 90,2 | 5 |
| 57012358 | keratin, type II cytoskeletal 73 [Rattus norvegicus] | 9,2 | 76,1 | 6 |
| 13591981 | lanosterol synthase [Rattus norvegicus] | 46,5 | 492,8 | 26 |
| 38014570 | Ldha protein [Rattus norvegicus] | 63,7 | 332,0 | 19 |
| 2981437 | lipocortin V [Rattus norvegicus] | 57,0 | 258,1 | 15 |
| 8393706 | L-lactate dehydrogenase A chain [Rattus norvegicus] | 66,3 | 447,7 | 24 |
| 16923952 | long-chain-fatty-acid--CoA ligase 3 [Rattus norvegicus] | 48,9 | 459,8 | 27 |
| 16758426 | long-chain-fatty-acid--CoA ligase 4 [Rattus norvegicus] | 59,0 | 524,1 | 30 |
| 103485105 | LPC acyltransferase [Rattus norvegicus] | 11,5 | 99,6 | 5 |
| 213688411 | lysophosphatidylcholine acyltransferase 1 [Rattus norvegicus] | 12,0 | 115,4 | 6 |
| 206205 | M2 pyruvate kinase [Rattus norvegicus] | 72,3 | 730,4 | 37 |
| 15100179 | malate dehydrogenase, cytoplasmic [Rattus norvegicus] | 44,3 | 172,0 | 11 |
| 82654202 | methyltransferase-like protein 7A [Rattus norvegicus] | 32,0 | 129,8 | 7 |
| 13540689 | moesin [Rattus norvegicus] | 30,0 | 236,1 | 17 |
| 6981236 | myosin-9 [Rattus norvegicus] | 36,5 | 1203,2 | 61 |
| 20302049 | NADH-cytochrome b5 reductase 3 [Rattus norvegicus] | 66,8 | 324,8 | 18 |
| 8394272 | nuclear migration protein nudC [Rattus norvegicus] | 24,4 | 119,6 | 7 |
| 161760644 | nucleolin [Rattus norvegicus] | 15,8 | 228,1 | 9 |
| 7242160 | nucleophosmin [Rattus norvegicus] | 26,4 | 88,2 | 6 |
| 77404363 | nucleosome assembly protein 1-like 1 [Rattus norvegicus] | 13,1 | 116,7 | 6 |
| 149061640 | patatin-like phospholipase domain containing 2 (predicted), isoform CRA_c [Rattus norvegicus] | 42,6 | 220,6 | 13 |
| 189095277 | patatin-like phospholipase domain-containing protein 2 [Rattus norvegicus] | 42,7 | 256,0 | 15 |
| 55742862 | perilipin-2 [Rattus norvegicus] | 65,9 | 435,5 | 21 |
| 16923958 | peroxiredoxin-1 [Rattus norvegicus] | 49,8 | 207,0 | 11 |
| 40254752 | phosphoglycerate kinase 1 [Rattus norvegicus] | 77,0 | 439,5 | 25 |
| 16757984 | phosphoglycerate mutase 1 [Rattus norvegicus] | 59,9 | 198,2 | 12 |
| 38181543 | Pkm2 protein [Rattus norvegicus] | 70,4 | 628,8 | 31 |
| 62078699 | probable saccharopine dehydrogenase [Rattus norvegicus] | 53,9 | 290,8 | 16 |
| 11693142 | proliferating cell nuclear antigen [Rattus norvegicus] | 44,8 | 158,8 | 9 |
| 51948384 | proliferation-associated protein 2G4 [Rattus norvegicus] | 23,1 | 132,7 | 8 |
| 61098214 | proteasome activator complex subunit 1 [Rattus norvegicus] | 35,8 | 144,7 | 9 |
| 6981324 | protein disulfide-isomerase [Rattus norvegicus] | 54,9 | 390,5 | 25 |
| 8393322 | protein disulfide-isomerase A3 precursor [Rattus norvegicus] | 47,9 | 375,8 | 22 |
| 16758712 | protein disulfide-isomerase A4 precursor [Rattus norvegicus] | 24,0 | 213,0 | 15 |
| 52345385 | protein disulfide-isomerase A6 [Rattus norvegicus] | 30,8 | 153,2 | 9 |
| 16757994 | pyruvate kinase isozymes M1/M2 [Rattus norvegicus] | 62,9 | 648,8 | 34 |
| 40254781 | rab GDP dissociation inhibitor beta [Rattus norvegicus] | 35,1 | 222,0 | 13 |
| 40804379 | radixin [Rattus norvegicus] | 11,2 | 110,8 | 8 |
| 58865970 | RAN GTPase activating protein 1 [Rattus norvegicus] | 23,0 | 143,8 | 8 |
| 14249144 | ras-related protein Rab-11B [Rattus norvegicus] | 46,8 | 176,2 | 10 |
| 16758368 | ras-related protein Rab-14 [Rattus norvegicus] | 37,7 | 111,2 | 8 |
| 121583768 | ras-related protein Rab-5B [Rattus norvegicus] | 47,9 | 128,6 | 8 |
| 347800697 | ras-related protein Rab-5C [Rattus norvegicus] | 58,8 | 176,0 | 9 |
| 149061923 | rCG48611, isoform CRA_b [Rattus norvegicus] | 48,7 | 115,8 | 7 |
| 149043692 | rCG60576, isoform CRA_a [Rattus norvegicus] | 53,5 | 541,0 | 30 |
| 55824765 | Serpinh1 protein [Rattus norvegicus] | 44,6 | 257,1 | 15 |
| 20302113 | stress-induced-phosphoprotein 1 [Rattus norvegicus] | 43,8 | 347,3 | 23 |
| 33414505 | T-complex protein 1 subunit delta [Rattus norvegicus] | 30,6 | 266,0 | 15 |
| 51890219 | T-complex protein 1 subunit epsilon [Rattus norvegicus] | 19,8 | 146,9 | 9 |
| 51858886 | Tra1 protein [Rattus norvegicus] | 31,9 | 343,9 | 19 |
| 42476292 | transaldolase [Rattus norvegicus] | 30,0 | 205,2 | 11 |
| 17865351 | transitional endoplasmic reticulum ATPase [Rattus norvegicus] | 41,7 | 490,1 | 27 |
| 12018252 | transketolase [Rattus norvegicus] | 42,8 | 360,8 | 22 |
| 14134101 | tropomyosin alpha isoform [Rattus norvegicus] | 22,2 | 50,0 | 6 |
| 29336093 | tropomyosin alpha-3 chain isoform 2 [Rattus norvegicus] | 26,6 | 116,6 | 8 |
| 6981672 | tropomyosin alpha-4 chain [Rattus norvegicus] | 26,6 | 119,4 | 10 |
| 20853961 | truncated alpha-actinin [Rattus norvegicus] | 7,2 | 107,7 | 6 |
| 38454226 | tumor protein D54 [Rattus norvegicus] | 62,7 | 227,9 | 13 |
| 57012432 | type I keratin KA11 [Rattus norvegicus] | 12,8 | 111,8 | 7 |
| 71043618 | tyrosyl-tRNA synthetase, cytoplasmic [Rattus norvegicus] | 31,3 | 241,5 | 15 |
| 13786146 | UDP-glucose 6-dehydrogenase [Rattus norvegicus] | 40,2 | 259,0 | 15 |
| 14389299 | vimentin [Rattus norvegicus] | 59,9 | 431,1 | 26 |
| 149031250 | vinculin (predicted), isoform CRA_a [Rattus norvegicus] | 36,3 | 614,5 | 35 |
|  |  |  |  |  |
